# The diagnostic value of dual-layer CT in the assessment of lymph nodes in lymphoma patients with PET/CT as a reference standard

**DOI:** 10.1038/s41598-023-45198-w

**Published:** 2023-10-26

**Authors:** Zhengwu Tan, Heng Mei, Chunxia Qin, Xiao Zhang, Ming Yang, Lan Zhang, Jing Wang

**Affiliations:** 1 Department of Radiology, Union Hospital, Tongji Medical College, Huazhong University of Science and Technology, No 1277, Jiefang Avenue, Wuhan, Hubei China; 2 Hubei Province Key Laboratory of Molecular Imaging, Wuhan, Hubei China; 3 Institute of Hematology, Union Hospital, Tongji Medical College, Huazhong University of Science and Technology, Wuhan, 430022 China; 4 Department of Nuclear Medicine, Union Hospital, Tongji Medical College, Huazhong University of Science and Technology, Wuhan, 430022 China; 5 Hubei Key Laboratory of Molecular Imaging, Union Hospital, Tongji Medical College, Huazhong University of Science and Technology, Wuhan, 430022 China

**Keywords:** Computed tomography, Whole body imaging, Lymphoma

## Abstract

This study aimed to evaluate the diagnostic performances of dual-layer CT (DLCT) for the identification of positive lymph nodes (LNs) in patients with lymphoma and retrospectively included 1165 LNs obtained by biopsy from 78 patients with histologically proven lymphoma, who underwent both pretreatment DLCT and 18F-fluorodeoxyglucose positron emission tomography/computed tomography (18F-FDG PET/CT). According to 18F-FDG PET/CT findings as a reference standard, cases were categorized into the LN-negative and LN-positive groups. LNs were then randomly divided at a ratio of 7:3 into the training (n = 809) and validation (n = 356) cohorts. The patients’ clinical characteristics and quantitative parameters including spectral curve slope (λ_HU_), iodine concentration (IC) on arterial phase (AP) and venous phase (VP) images were compared between the LN-negative and LN-positive groups using Chi-square test, t-test or Mann–Whitney U test for categorical variables or quantitative parameters. Multivariate logistic regression analysis with tenfold cross-validation was performed to establish the most efficient predictive model in the training cohort. The area under the curve (AUC) was used to evaluate the diagnostic value of the predictive model, and differences in AUC were determined by the DeLong test. Moreover, the predictive model was validated in the validation cohort. Repeatability analysis was performed for LNs using intraclass correlation coefficients (ICCs). In the training cohort, long diameter (LD) had the highest AUC as an independent factors compared to other parameter in differentiating LN positivity from LN negativity (*p* = 0.006 to *p* < 0.001), and the AUC of predictive model jointly involving LD and λ_HU_-AP was significantly elevated (AUC of 0.816, *p* < 0.001). While the AUC of predictive model in the validation cohort was 0.786. Good to excellent repeatability was observed for all parameters (ICC > 0.75). The combination of DLCT with morphological and functional parameters may represent a potential imaging biomarker for detecting LN positivity in lymphoma.

## Introduction

In recent years, the incidence of lymphoma (e.g., Hodgkin’s lymphoma and non-Hodgkin’s lymphoma) has dramatically increased by 12.9% and 54.8% between 2005 and 2015, respectively, as revealed by an analysis of global cancer incidence^1^, and lymphoma is estimated to account for 3–4% of all cancers worldwide^2^. Accurate staging is of great value because of its prognostic and therapeutic implications. Lymphoma staging predominantly relies on the Lugano classification system^3^, which takes into account the number of involved sites, the type of lesions (nodal or extra-nodal, mainly involving lymph nodes), and disease distribution based on 18F-fluorodeoxyglucose (FDG) positron emission tomography (PET)/computed tomography (CT) (18F-FDG PET/CT), computed tomography (CT), and magnetic resonance imaging (MRI). 18F-FDGPET/CT is a basic examination preceding therapy in the staging process and following CT. However, factors limiting 18F-FDGPET/CT use include elevated cost, radioisotope use and differences in the geographic distribution of large tertiary care centers in many countries^4–6^. Diffusion-weighted imaging (DWI) shows a significant agreement with PET/CT for initial evaluation, staging, and treatment response assessment in lymphoma, but is time-consuming^7^. Thus, in clinical practice, CT remains the most commonly applied imaging modality for routine staging of lymphoma^8,9^. As plain CT tends to identify nodes as hilar structures and cannot well differentiate the boundary between single large nodes and normal structures, contrast-enhanced CT is employed to accurately measure the node size.

In the CT presentation of lymphoma, conventional enhancement CT can only examine lymph nodes based on morphology, with short diameter (> 10 mm) or long diameter (> 15 mm) as a diagnostic criterion in CT^10^ indicating positive lymph nodes according to CT image evaluation in the Lugano and Ann Arbor classification systems^3,8^. Meanwhile, positive lymph nodes (short diameter < 10 mm or long diameter < 15 mm) also show high uptake in 18F-FDG PET/CT imaging^11^, which is easily misdiagnosed on conventional enhancement CT images. Textural and radiomic features derived from CT may improve the diagnostic performance for the disease compared to morphological features obtained by CT^12^, which is affected by a variety of factors, including CT acquisition parameters, reconstruction setting and image quality, which may result in compromised reliability and data inconsistency, requiring massive preprocessing and segmentation of images, computational resources and time, with lack of standardization, repeatability, and reproducibility^13–18^.

Dual-energy CT (DECT) has enabled novel applications in clinical practice, including virtual monochromatic imaging (VMI), iodine concentration (IC) mapping and spectral curve slope (λ_HU_), which have shown promise for preoperative diagnosis of metastatic lymph nodes in patients with various types of cancer^19–21^ because DECT provides functional parameters versus morphological indexes obtained by conventional CT. However, the importance of quantitative parameters in the diagnosis of LNs in patients with lymphoma remains elusive. Dual-layer CT (DLCT) as a short scanning time and a fast post-processing process, and lower radiation dose has been further achieved by utilizing the energy spectrum CT technology; in addition, DLCT datasets can be obtained in conventional scanning and the “retrospective analysis” function can be utilized subsequently for energy spectrum data^22,23^. Thus, the present study aimed to assess the diagnostic value of spectral DLCT in differentiating LN positivity from LN-negativity based on PET/CT as a reference standard.

## Results

### Participants’ demographic characteristics

**Figure 1:**
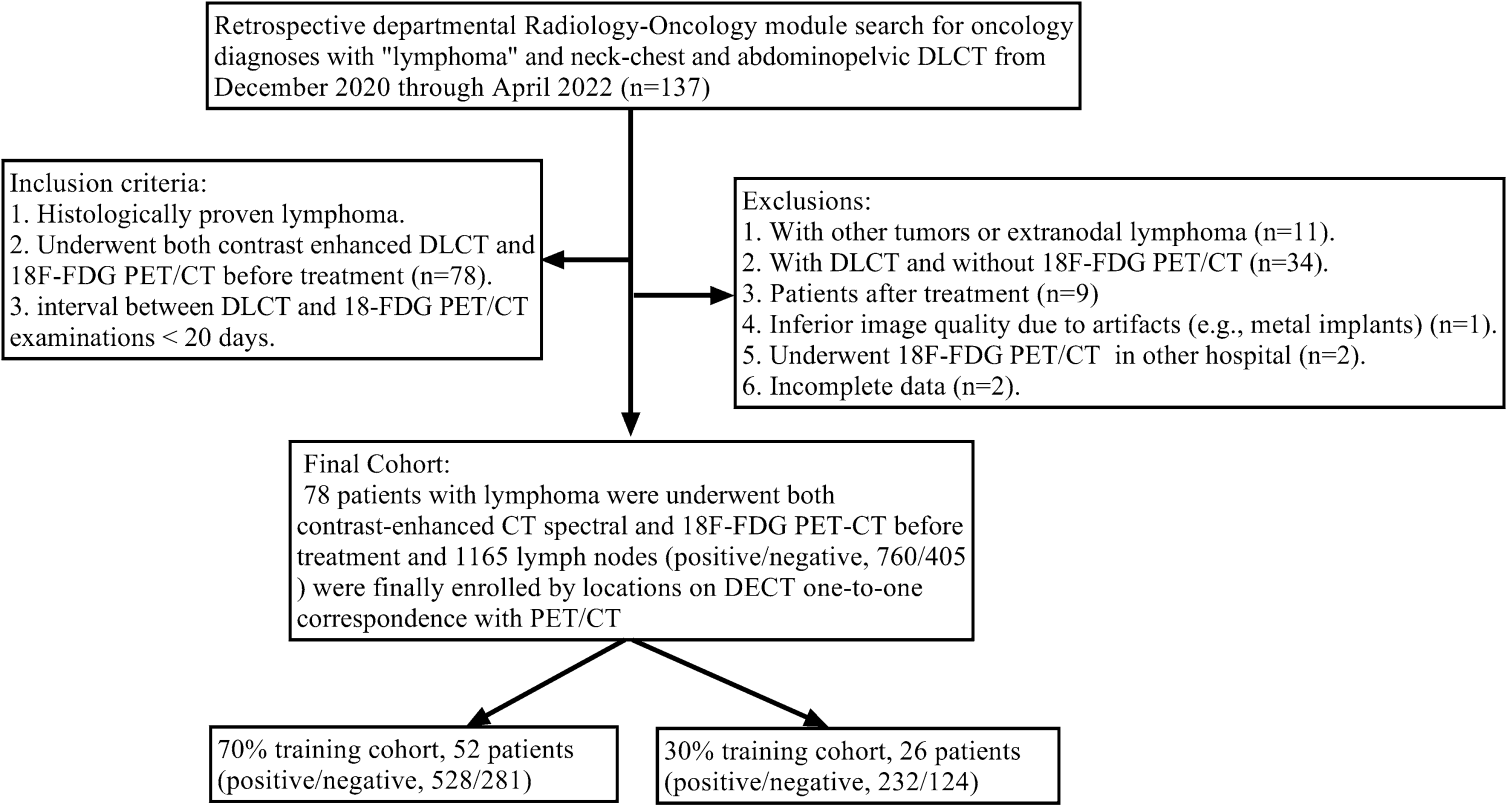
Study flowchart. DLCT: dual-layer CT; 18F-FDG PET/CT: fluorodeoxyglucose positron-emission-tomography/CT.

A total of 78 patients (43 males and 35 females; 53.95 ± 13.30 years old) underwent DLCT and PET/CT with an interval time of 5.79 ± 5.76 days. Based on a 7:3 ratio, 1165 LNs from 78 patients were randomly divided into the training (n = 809, 52 patients) and validation (n = 356, 26 patients) cohorts (shown in Fig. 1). There were 52 patients with 528 positive LNs (SUV_max_: 8.58 ± 6.29) and 281 negative LNs (SUV_max_: 1.39 ± 0.5) in the training cohort, and 26 patients with 232 positive LNs (SUV_max_: 7.69 ± 5.98) and 124 negative LNs (SUV_max_: 1.64 ± 0.65) in the validation cohort. The patients’ demographic characteristics, types of lymphoma and nodal sites in the training and validation cohorts are summarized in Table 1.

**Table 1:** Patients’ demographic and clinical data.

Characteristic	Total	Training cohort	Validation cohort
Number of cases	78	52	26
Gender			
Male	43	32	11
Female	35	20	15
Age (years)	53.95 ± 13.30	51.99 ± 11.57	58.79 ± 14.64
Range	13–83	30–83	13–83
Type of lymphoma			
DLBCL	45	28	17
FL	11	9	2
NK-TL	5	4	1
MCL	5	2	3
AILD TCL	3	2	1
PTCL	3	3	0
Other	6	4	2
Nodal longest-axis diameter (positive/negative)	760/405	528/281	232/124
Nodal sites (positive/negative)			
Cervical	214/98	131/57	83/41
Axillary	98/42	74/26	24/16
Mediastinal	99/48	65/34	34/14
Para-aortic	164/95	119/69	45/26
Mesenteric	32/22	32/22	0/0
Para-iliac	81/46	56/34	25/12
Inguinal femoral	72/54	51/39	21/15

*DLBCL* Diffuse large B-cell lymphoma, *FL* Follicular lymphoma, *NKTL* Natural killer/T-cell lymphoma, *MCL* Mantle cell lymphoma, *AILD TCL* Angioimmunoblastic lymphadenopathy like T-cell lymphoma, *PTCL* Peripheral T-cell lymphoma.

### Repeatability analysis

For all parameters (SUV_max_, LD, IC-AP, NIC-AP, λ_HU_-AP, IC-VP, NIC-VP, and λ_HU_-VP), good to excellent inter-reader measurement repeatability was observed for DLCT imaging parameters, with ICC values higher than 0.75 (ICCs of 0.965, 0.961, 0.995, 0.992, 0.98, 0.993, 0.763, and 0.97, respectively).

### Clinical characteristics and quantitative parameters of LNs

**Figure 2:**
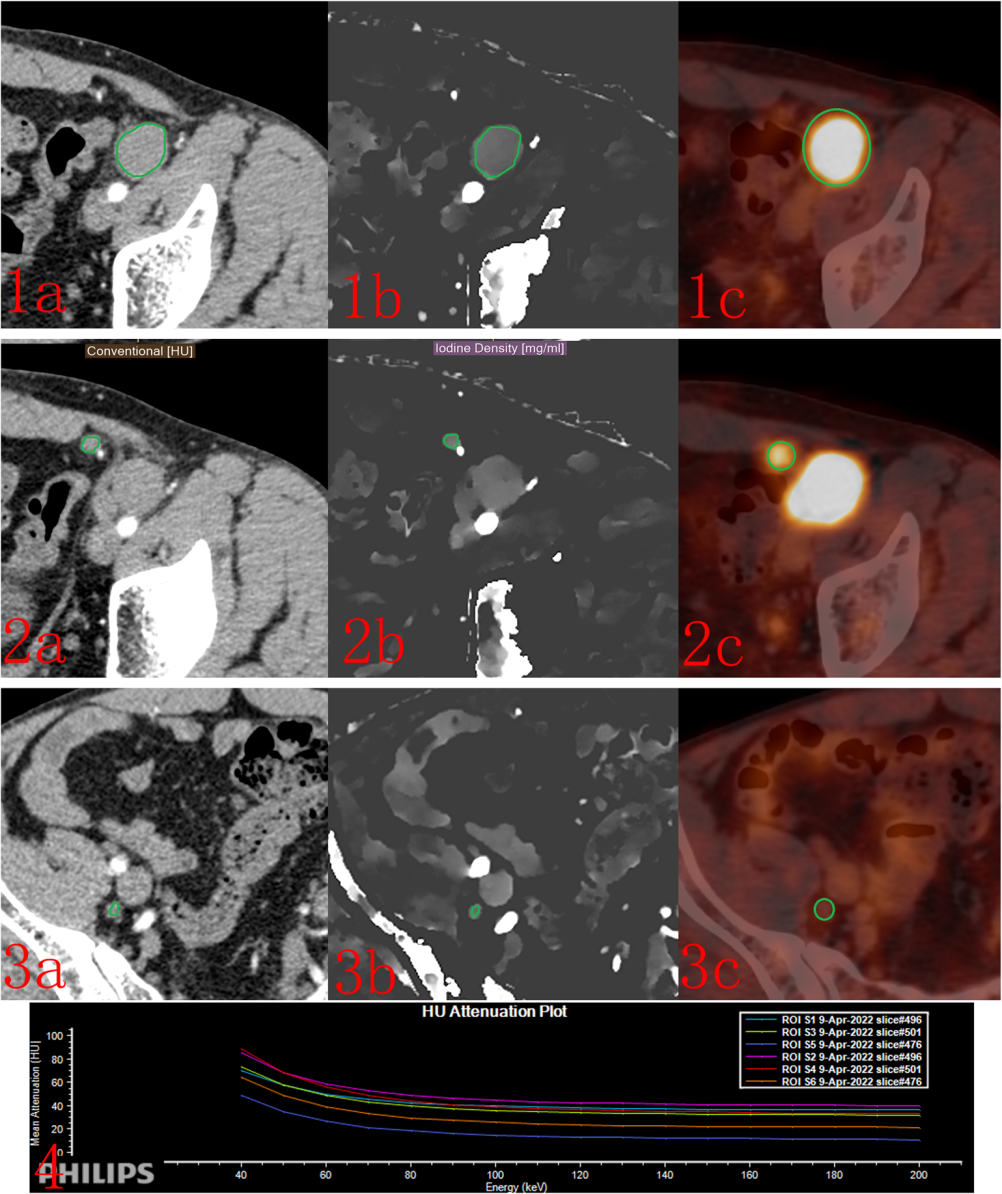
CT images of a 43-year-old male patient with lymphoma. (1a, 2a, 3a) Conventional images of para-iliac LNs in the AP. (1b, 2b, 3b) Iodine density images in the same layer as AP scans. (1c, 2c, 3c) SUV_max_ in 18F-FDG PET/CT images. (4) Spectral curves in the AP and VP (λ_HU_-AP: ROI S1, S3, S5; λ_HU_-VP: S2, S4, S6). For LNs in region 1: LD = 20.60 mm, SUV_max_ = 20.1, IC-AP = 0.49 mg/mL, NIC-AP = 0.08 mg/mL, λ_HU_-AP = 0.98, IC-VP = 0.67 mg/mL, NIC-VP = 0.22 mg/mL, and λ_HU_-VP = 1.34. For LNs in region 2: LD = 8.2 mm, SUV_max_ = 6.70, IC-AP = 0.43 mg/mL, NIC-AP = 0.07 mg/mL, λ_HU_-AP = 0.87, IC-VP = 0.57 mg/mL, NIC-VP = 0.19 mg/mL, andλ_HU_-VP = 0.72. For LNs in region 3: LD = 8.0 mm, SUV_max_ = 1.30, IC-AP = 0.45 mg/mL, NIC-AP = 0.08 mg/mL, λ_HU_-AP = 0.90, IC-VP = 0.51 mg/mL, NIC-VP = 0.17 mg/mL, and λ_HU_-VP = 1.03. The SUV_max_ of mediastinal blood pool was 2.3. LNs in regions 1 and 2 were positive. LNs in region 3 were negative. AP: arterial phase; VP: venous phase; LD: long-axis diameter; NIC: normalized iodine concentration; λ_HU_: spectral curve slope; SUV_max_: maximum standardized uptake value.

In the training cohort, four clinical characteristics, including gender, age, type of lymphoma and nodal site, showed no significant differences between the LN-positive and LN-negative groups (*p* = 0.149 to 0.790), while DLCT imaging parameters, including LD, IC, NIC, and λ_HU_ on both AP and VP images were significantly higher in the LN-positive group compared with the LN-negative group in the training and validation cohorts (Table 2 and Fig. 2) (*p* = 0.002 to < 0.001).

**Table 2:** Clinical characteristics and quantitative DLCT parameters of LNs in lymphoma patients.

Features	Total			Training cohort			Validation cohort		
Parameter	LN-positive group (760)	LN-negative group (405)	*p*	LN-positive group (528)	LN-negative group (281)	*p*	LN-positive group (232)	LN-negative group (124)	*p*
SUV_max_	8.30 ± 6.21	1.47 ± 0.56	< 0.001	8.58 ± 6.29	1.39 ± 0.5	< 0.001	7.69 ± 5.98	1.64 ± 0.65	< 0.001
Gender						0.317			0.139
Age (years)						0.736			0.916
Type of lymphoma						0.790			0.057
Nodal site						0.149			0.813
LD (mm)	15.79 ± 8.29	10.63 ± 2.69	< 0.001	15.85 ± 8.2	10.6 ± 2.7	< 0.001	15.68 ± 8.5	10.7 ± 2.7	< 0.001
IC-AP (mg/mL)	1.38 ± 6.23	0.78 ± 0.62	< 0.001	1.49 ± 7.46	0.79 ± 0.67	< 0.001	1.13 ± 0.51	0.76 ± 0.49	< 0.001
NIC-AP	0.17 ± 0.69	0.10 ± 0.09	< 0.001	0.18 ± 0.83	0.11 ± 0.1	< 0.001	0.14 ± 0.07	0.09 ± 0.06	< 0.001
λ_HU_-AP	2.31 ± 1.22	1.49 ± 1.39	< 0.001	2.34 ± 1.27	1.51 ± 1.47	< 0.001	2.24 ± 1.07	1.46 ± 1.2	< 0.001
IC-VP (mg/mL)	1.23 ± 0.50	1.03 ± 0.59	< 0.001	1.23 ± 0.53	1.04 ± 0.63	< 0.001	1.22 ± 0.44	1.01 ± 0.46	< 0.001
NIC-VP	0.33 ± 0.13	0.30 ± 0.17	< 0.001	0.34 ± 0.13	0.31 ± 0.18	0.002	0.32 ± 0.13	0.27 ± 0.13	0.001
λ_HU_-VP	2.46 ± 1.03	2.01 ± 1.30	< 0.001	2.48 ± 1.07	2.02 ± 1.37	< 0.001	2.43 ± 0.92	1.98 ± 1.15	< 0.001

*DLCT* Dual-layer computed tomography, *LN* Lymph node, *LD* Longest-axis diameter, *SUV*_*max*_ Maximum standardized uptake value, *NIC* Normalized iodine concentration, *AP* Arterial phase, *λ*_*HU*_ Spectral curve slope, *VP* Venous phase.

### Predictive model establishment

LD and DLCT imaging parameters in the training cohort were selected by univariate and multivariate analyses, and two indexes were significant independent factors for identifying LN positivity in lymphoma, including LD (OR = 0.321, 95% confidence interval [CI] 1.252–1.395; *p* < 0.001) and λ_HU_-AP (OR = 1.874, 95% CI 1.461–2.405; *p* < 0.001) (Table 3).

**Table 3:** Independent predictors of positive lymph nodes in univariate and multivariate analyses in the training cohort.

Characteristics	Univariate analysis		Multivariate analysis	
	*p* value	OR (95% CI)	*p* value	OR (95% CI)
Gender	0.317	0. 860 (0.640–1.156)		
Age (years)	0.736	0. 998 (0.985–1.010)		
Type of lymphoma				
AILD TCL (as reference)	0.796			
DLBCL	0.151	2.120 (0.760–5.909)		
FL	0.514	1.254 (0.636–2.472)		
NK-TL	0.838	1.104 (0.429–2.840)		
MCL	0.895	1.051 (0.501–2.208)		
PTCL	0.571	1.273 (0.552–2.939)		
Other	0.577	1.272 (0.546–2.960)		
Nodal site				
Mesenteric (as reference)	0.154			
Cervical	0.692	0. 902 (0.542–1.502)		
Axillary	0.623	0. 862 (0.475–1.562)		
Mediastinal	0.201	1.489 (0.809–2.739)		
Para-aortic	0.206	0. 684 (0.380–1.232)		
Para-iliac	0.486	1.202 (0.716–2.019)		
Inguinal femoral	0.433	0. 761 (0.384–1.507)		
LD (mm)	< 0.001	1.300 (1.236–1.367)	< 0.001	0.321 (1.252–1.395)
IC-AP (mg/mL)	< 0.001	2.639 (2.038–3.417)	0.409	1.234 (0.749–2.032)
NIC-AP	< 0.001	239.239 (37.235–1537.122)	0.423	0.186 (0.003–11.394)
λ_HU_-AP	< 0.001	1.623 (1.434–1.837)	< 0.001	1.874 (1.461–2.405)
IC-VP (mg/mL)	< 0.001	1.880 (1.438–2.458)	0.103	0.0002 (0–5.386)
NIC-VP	0.002	4.855 (1.794–13.137)	0.318	0.317 (0.033–3.021)
λ_HU_-VP	< 0.001	1.392 (1.222–1.584)	0.102	67.095 (0.435–10,339.244)

### Evaluation of the diagnostic performances of the predictive combination model

In the training cohort, LD and DLCT imaging parameters with statistical significance in multivariate analysis were selected to establish a predictive combination model by multivariate logistic regression analysis with tenfold cross-validation, in which LD and λ_HU_-AP were combined.

**Figure 3:**
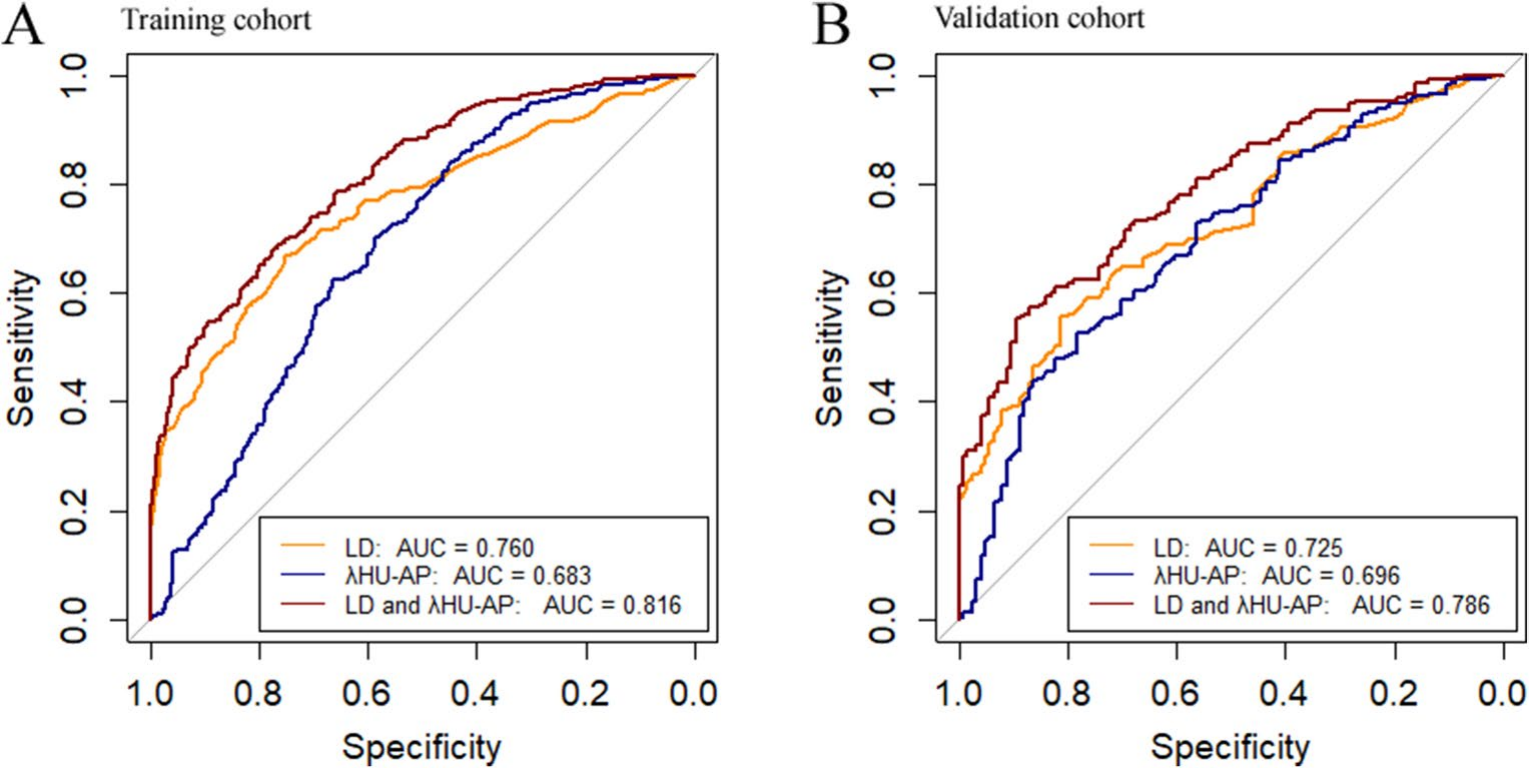
Diagnostic efficiencies of DLCT imaging parameters and the predictive combination model using receiver operating characteristic curve analysis in the training and validation cohorts.

In the training cohort, the diagnostic performances of all individual quantitative parameters and the predictive combination model are shown in Table 4. LD, IC, NIC, and λ_HU_ on AP images had good performances with AUCs of 0.760, 0.682, 0.669, and 0.683, respectively (all *p* < 0.001); LD had the highest AUC of 0.760, with a cutoff of 11.7 mm, which was higher compared with those of individual spectral parameters (IC, NIC, and λ_HU_-AP; *p* = 0.005, 0.001, 0.006, respectively). The AUCs of DLCT parameters on AP images were significantly superior to those of VP images (all, *p* < 0.001). The AUC of the predictive model jointly involving LD and λ_HU_-AP (AUC = 0.816) in the training cohort was significantly higher compared with that of LD (0.760; *p* < 0.001) or λ_HU_-AP (0.683; *p* < 0.001) alone, in differentiating LN positivity from LN-negativity. In the validation cohort, the AUC of combined LD and λ_HU_-AP was 0.786, which was also significantly higher than the AUC of LD (0.725; *p* < 0.001) or λ_HU_-AP (0.696; *p* = 0.001) alone. These data are summarized in Table 4 and Fig. 3.

**Table 4:** Comparison of the diagnostic efficiencies of DLCT parameters for lymph node assessment.

	AUC	Threshold	Accuracy (%)	Sensitivity (%)	Specificity (%)	PPV (%)	NPV (%)	*P*
Training cohort								
Single parameter								
LD (mm)	0.760	11.70	69.59	66.67	75.09	83.41	54.52	< 0.001
Lugano criterion		15.00	59.50	43.75	87.19	89.53	45.20	< 0.001
IC-AP (mg/mL)	0.682	0.56	70.33	84.09	44.48	74.00	59.81	< 0.001
NIC-AP	0.669	0.08	68.60	78.41	50.18	74.73	55.30	< 0.001
λ_HU_-AP	0.683	1.15	70.46	83.90	45.20	74.21	59.91	< 0.001
IC-VP (mg/mL)	0.601	0.65	69.84	89.77	32.38	71.38	62.75	< 0.001
NIC-VP	0.567	0.18	68.85	91.10	27.05	70.12	61.80	< 0.001
λ_HU_-VP	0.602	1.32	69.84	89.77	32.38	71.38	62.75	< 0.001
Predictive model								
LD and λ_HU_-AP	0.816	0.65	71.08	67.80	77.22	84.83	56.07	< 0.001
Validation cohort								
Single parameter								
LD (mm)	0.725	12.50	64.60	55.60	81.45	84.87	49.51	< 0.001
λ_HU_-AP	0.696	2.10	61.52	52.59	78.23	81.88	46.86	< 0.001
Predictive model								
LD and λ_HU_-AP	0.786	–	67.70	64.66	73.39	81.97	52.60	< 0.001

*DLCT* Dual-layer computed tomography, *AUC* Area under the curve, *PPV* Positive predictive value, *NPV* Negative predictive value, *LD* Longest-axis diameter, *NIC* Normalized iodine concentration, *AP* Arterial phase, *λ*_*HU*_ Spectral curve slope, *VP* Venous phase.

ROC analysis of SUV_max_ was not available as it was used as a reference standard for defining lymph nodes as negative or positive.

### LD of the LN-positive group with a cutoff of 15 mm

In the training cohort, among the 809 LNs examined, based on the Lugano classification system and considering the commonly applied threshold of 15 mm for LD to indicate LN positivity for lymphomatous involvement on CT, an accuracy of 59.50%, a sensitivity of 43.75%, a specificity of 87.19%, a positive predictive value of 89.53% and a negative predictive value of 45.20% were found in the identification of LN positivity (Table 4).

## Discussion

In the present study, the diagnostic performances of spectral quantitative parameters in differentiating positive LNs from negative LNs in lymphoma patients before treatment were examined. In multivariate analysis, LD and λ_HU_-AP were independent risk factors for LN positivity in lymphoma. Therefore, we developed and validated a novel predictive model combining LD and λ_HU_-AP, which had good accuracy in both training and validation cohorts for pre-treatment identification of positive LNs in patients with lymphoma. The combination of LD and λ_HU_-AP outperformed LD or λ_HU_-AP used alone in the training cohort for assessing the involvement of LNs in lymphoma, with the highest AUC of 0.816. This predictive model with an AUC of 0.786 was also well validated in the validation cohort.

Recently, quantitative parameters such as λ_HU_, IC and NIC derived from spectral imaging data on DLCT scans have been shown to be advantageous for diagnosis and nodal staging in patients with rectal cancer^24^, lung cancer^25^, cervical lymphadenopathy^20^, and gastric cancer^19^. To the best of our knowledge, this is the first study that used quantitative parameters derived from spectral DLCT to evaluate nodal involvement in lymphoma considering 18F-FDG PET/CT as a reference standard. Meanwhile, a significant correlation between metabolic activity (SUV_max_) as assessed by 18F-FDGPET/CT and iodine concentration as evaluated by DLCT was reported by Gehling et al.^26^. In the current study, image parameters obtained by DLCT were significantly higher in the LN-positive group compared with the LN-negative group, and exhibited moderate diagnostic performances in the training cohort. LD with an AUC of 0.760 was the most effective single factor discriminating positive LNs from negative LNs, which is consistent with an AUC of 0.774 for LD in differentiating lymph nodes with and without metastasis in patients with gastric cancers, as previously reported by Zhou et al.^27^. As shown above, LD (OR = 0.321) and λ_HU_-AP (OR = 1.847) in the training cohort were independent risk factors for LN positivity in lymphoma by multivariate analysis. Additionally, the combination of both morphology reflected by LD and spectral function reflected by λ_HU_-AP resulted in a significantly improved accuracy of 71.08% compared with LD and λ_HU_-AP alone (69.59% and 70.46%, respectively) and significantly increased diagnostic efficiency (AUC = 0.816) compared with each single factor (AUC = 0.760 and 0.683, respectively) in the training cohort; similar results were obtained in the validation cohort (AUC = 0.786), which may be due to combining the advantages of both factors that could allow for a better evaluation of lesions in lymph nodes and promote the development of new diagnostic imaging techniques in lymphoma. Similar improvement of performance using spectral combination was also reported in a study by Gao et al.^25^, in which the combination of λ_HU_ and short-axis diameter was also significantly preferred to single parameters in lung cancer. Liu et al.^24^ also reported that utilizing the combination of quantitative parameters derived from DECT and short-axis diameter to assess metastatic and non-metastatic LNs in rectal cancer improved accuracy and AUC to 82.9% and 0.82 from 61.0% and 0.65 obtained with NIC and 65.7% and 0.81 obtained with short-axis, respectively. Chen et al.^28^ reported AUCs for a DECT-based model of 0.842 and 0.848 in the training and validation cohorts, respectively, for preoperative prediction of lymph node metastasis in papillary thyroid carcinoma; these AUCs were slightly higher than those of this study, which might be derived from the different types and tissue characteristics of lymph node lesions^20^.

The staging of lymphoma by CT is mainly based on the morphological features of LNs. Peripheral nodes are considered abnormal with LD > 20 mm, while nodes with LDs between 10 and 20 mm still need to be examined for potential FDG/PET-avidity^29^. A 10–15 mm cutoff for LD is commonly used to discriminate between “probably benign LNs” and “probably malignant LNs”, although this is arbitrary. Our results showed that a 15 mm cut-off for LD, according to CT image evaluation in the Lugano classification system, yielded a sensitivity of 43.75%, while its accuracy was only 59.50% in the training cohort. Kwee et al.^30^ reported an accuracy of 66.6% for lymphoma staging based on a short-axis diameter exceeding 10 mm on CT images. However, considering all lymph nodes with a cutoff of 11.7 mm, the accuracy was improved to 69.59% and a sensitivity of 66.67% was obtained. Although a high number of small nodes may be considered suspiciously malignant in some clinical circumstances, in the present study, LNs with LD < 10 mm were involved in lymphomas, corroborating a previous study^31^. Therefore, considering LD as an independent risk factor, a 12 mm cutoff for LD may improve diagnostic performance for identifying positive lymph nodes in lymphoma based on CT in clinical practice.

In this study, LDs greater than 8 mm were included because SUV is of limited value when assessing relatively small lymph nodes^32^, and the partial volume effect also occurs in small lesion quantification by CT. In this situation, a subjective assessment is more appropriate for deciding whether contrast uptake has increased above that of the adjacent soft tissue. Therefore, we considered in this assessment lymph nodes both with higher 18F-FDG uptake relative to surrounding background on visual analysis and SUV_max_ greater than the mediastinal blood pool (DC ≥ 3) and subsequent follow-up imaging as positive^33^. We found that CT spectral parameters in the AP were superior to those in the VP in this study, which may be related to the fact that IC-AP reflects functional capillary density^34^. Spectral CT studies of metastatic lymph nodes in gastric cancer by Zhou et al.^27^ and in colon cancer by Yang et al.^35^ reported that CT parameters in the AP were superior to those in the VP, while Li et al.^19^ reported opposite results. Chen et al.^28^ also reported that λ_HU_ in the AP were superior to those in the VP for predicting lymph node metastasis in papillary thyroid carcinoma patients, corroborating the current results. In the current study, λ_HU_ was slightly preferable to iodine mapping because λ_HU_ is helpful for the characterization of specific tissue types and compositional analysis to obtain diagnostic evaluation based on different tissues^36^. Both AP and VP scans were obtained to determine which parameters are better in the AP or VP, and to better evaluate the involvement of different organs in lymphoma patients. We excluded extranodal lymphomas and lymphoma lesions involving extranodal organs, because lymphomas can involve different extranodal organs, which is a too complex situation due to the heterogeneous nature and complex components of tumors and the normal tissue.

There were several limitations in the present study. Firstly, this was a retrospective single-center study with no external validation; therefore, the current findings need to be further validated by external datasets or multicenter studies. Secondly, the sample size was not large enough and included many lymphoma types such as common DLBCL and uncommon lymphomas, although this is consistent with the epidemiology of lymphoma. However, there were no significant differences in pathological type, LN location, age group, and gender between the LN-positive and LN-negative groups in the training cohort, which is worthy of further investigation. Thirdly, we used18F-FDG PET/CT as a reference standard and performed DLCT-18F-FDG PET/CT for one-to-one matching of LNs, but no histopathological reference standard was used for the involvement of LN sites; as lymphoma is generally diffuse, surgical exploration of all possibly affected LN sites in histological examination is impossible for ethical and practical reasons. Finally, we did not segment lymph nodes and then assessed them based on 3D target volumes. In this study, positive lymph nodes in lymphoma were identified via routine review by 2 nuclear medicine physicians and 2 radiologists, and quantitative parameters were measured independently by two radiologists with good to excellent repeatability. In future research, automatic 3D segmentation will be performed to measure the maximum CT parameters of lymph nodes and match them with SUV_max_.

## Conclusion

In conclusion, this study showed that the combination of LD and λ_HU_-AP derived from DLCT has a high diagnostic performance in assessing LN involvement in lymphoma. DLCT could be developed as an examination tool combining both morphology and function for the identification or screening of positive LNs in lymphoma patients because of the relatively lower cost and lack of radioisotope radiation associated with CT compared with 18F-FDG PET/CT.

## Methods

### Participants

This retrospective, single-center study was approved by the Institutional Review Board of our Hospital. The requirement for written informed consent was waived due to the retrospective nature of this study. The demographic and clinical data of consecutive patients who underwent DLCT were collected between December 2020 and April 2022. The study workflow is shown in Fig. 1. Inclusion criteria were: ① histologically proven lymphoma by biopsy; ② contrast enhanced DLCT and18F-FDG PET/CT before treatment (n = 78); ③ time interval between DLCT and 18F-FDG PET/CT examinations < 20 days. Exclusion criteria were: ① other tumors or extranodal lymphoma (n = 11); ② DLCT and no18F-FDG PET/CT (n = 34); ③previous cancer treatment (n = 9); ④ low image quality due to artifacts (e.g., metal implants) (n = 1); ⑤ 18F-FDG PET/CT in another hospital (n = 2); ⑥ incomplete imagingdata (n = 2). Following these criteria, 59 of the 137 initially identified consecutive patients were excluded, resulting in the enrollment of a total of 78 patients in this study.

### Image acquisition

#### 18F-FDG PET/CT

All patients fasted for more than 6 h, with blood glucose ≤ 200 mg/dL before injection. 18F-FDG PET/CT was performed on a Discovery VCT 64-slice PET/CT scanner (GE Healthcare, Milwaukee, WI, USA) at almost 60 min after intravenous injection of 3.7 MBq/kg of 18F-FDG (mean, 64 min; range, 50–90 min; ± 7.8). 18F-FDG PET/CT imaging was performed in the 3D mode, covering vertex to mid thighs with 6–7 beds. 18F-FDG PET/CT images were reconstructed by the ordered subsets expectation maximization (OSEM) method. CT was performed according to a standard protocol with the following parameters: tube voltage, 120 keV; tube current, 110 mA; slice thickness, 3.75 mm. The mean PET/CT scanning time per patient was 20.4 min (range, 15.5–22.5 min; ± 1.4).

#### CT

CT scans of the neck, chest, abdomen, and pelvis were performed on a DLCT instrument (IQon® Spectral CT; Philips Healthcare, Amsterdam, The Netherlands). All patients underwent an intravenous injection of a non-ionic iodinated contrast agent (Ultravist 300; Schering, Berlin, Germany) before scanning. The amount of the CT contrast agent was adjusted according to weight (320 mgl/kg); injection into the antecubital vein used the fixed scan delay technology for a duration of 25–30 s. Scan delay after injection was set to 30 and 60 s, to depict the arterial phase (AP) and venous phase (VP), respectively. CT scan with an axial field of view (FOV) of 320—360 mm (according to body size) and a matrix of 512 × 512 was performed in the spiral mode under continuous acquisition at a tube voltage of 120 kV and a tube current of 138 mA (slice thickness, 1.5 mm; increment, 2.5 mm/s; rotation time, 0.5 s; pitch 1; collimation, 64 × 0.625; kernel). CT dose index was set to 32 mGy. Conventional images (CIs), virtual monoenergetic images (VMI), and iodine maps were reconstructed (Spectral B, iDose level 3; Philips Healthcare).

#### Determination of lymph nodes

Using 18F-FDG PET/CT as a reference standard, 18F-FDG PET/CT and CT images were retrospectively reviewed by 2 nuclear medicine physicians (7 and 5 years of experience in PET/CT, respectively) and 2 radiologists (7 and 5 years of experience in CT of the neck, thorax and abdomen, respectively), who identified LN positivity according to the following consensus Deauville criteria (DC) of the International Harmonization Project^37^: lymph nodes ① with higher 18F-FDG uptake than the mediastinal bloodpool (DC ≥ 3) and ② surrounding background on visual analysis of baseline-FDG-PET/CT images^3,26,38,39^ and ③ subsequent follow-up imaging^40,41^ indicating unequivocal diagnosis based on PET/CT. Discrepancies between CT and 18F-FDG PET/CT findings were assessed by an expert panel (radiologists and nuclear medicine physicians with 10 years of experience, respectively, with knowledge of clinical history, and each focal tracer uptake deviating from physiological distribution and background was regarded as suggestive of disease). Lymph nodes with equivocal diagnosis determined by the expert panel were excluded. Positive and negative LNs were annotated for quantitative analysis. Further information on the locations and sizes of the included lymph nodes is given in Table 1 and Fig. 2, respectively. Nodal sites were classified as the following regions: cervical, axillary, mediastinal (including hilar), para-aortic, mesenteric, para-iliac, and inguinal femoral. In case of positive LNs in these regions, a maximum number of 6 lymphoma lesions with different sizes were assessed, and negative LNs with less lesions than found in positive LNs were selected^3,26^. According to 18F-FDG PET/CT findings, 1165 LNs (760 positive and 406negative) from 78 patients were continuously enrolled and randomly divided into the training and validation cohorts at a ratio of 7:3.

### Data analysis

At a time interval of a month between annotated and quantitively analyzed LNs, the LD values of all LNs were independently measured by the 2 abovementioned radiologists, with experience in reporting CT using conventional CT images in the VP. LNs with LD > 8 mm, which are considered measurable^27,40^, were recorded and analyzed. All CT measurements were performed on a vendor-specific commercial spectral CT workstation (Philips Healthcare). Freehand regions of interest (ROIs) were manually delineated as large as possible in the solid component of target LNs to avoid necrotic or cystic areas, calcification sites, and hardening artifacts of bones on AP and VP images in the largest cross-sectional area. CT values on VMI (40/70 keV) and iodine concentration (IC) for each LN were calculated. Aortic IC was also measured by placing circular ROIs on the same slice, carefully avoiding calcified plaques secondary to atherosclerosis, and used to normalize the IC of the LN as NIC (NIC = IC_LN_/IC_aorta_). As the spectral curve exhibited smaller changes and differences at energy levels greater than 120 keV, the slope of the spectral curve λ_HU_ (λ_HU_ = (CT_40keV_–CT_70keV_)/30) was used in the present study^42^. DLCT parameters are explained in Table 5. Two radiologists independently measured LNs for ROI, for the evaluation of inter-reader repeatability. The average values measured by two radiologists were considered the mean values.

**Table 5:** The explanation of the parameters from DLCT biomarkers.

Parameter		Biomarker measured	Pathophysiological processes informed by biomarker	Units
IC		Use of substance decomposition to generate iodine maps, i.e., iodine-enhanced images from enhanced dual-energy CT images	Strong correlation with microvessel density and vascular endothelial growth factor to differentiate between enhancing and nonenhancing lesions, which improves visualization of hypervascular and hypovascular masses	Mg/mL
	IC-AP		Reflects in the functional capillary density	Mg/mL
	IC-VP		Reflects in the balance of blood supply and the late-phase retention of the contrast agent in the interstitial space	Mg/mL
	NIC-AP/NIC-VP	The lymph nodes iodine concentration was normalized to that of aorta at the same image level (IC_LN_/IC_aorta_)	Minimizes variations in contrast input function, patient’s hemodynamic status, patient’s body weight, and amount of tissue that iodine may distribute rather than absolute tumor iodine concentration, more likely to normalizes the technical and physiological variations, and compensate inter-subjective and intrasubjective variability	
λ_HU_	λ_HU_-AP/λ_HU_-VP	Energy spectral curves were obtained by setting a region of interest in the tissue and plotting the average CT value in virtual monochromatic images	Not only assesses differences in the attenuation of various tissues at distinct photon energies but also provides quantitative parameters reflecting the biological characteristics of specific tissue types and contributes to component analysis and differential diagnosis, for example tumors and different normal tissues	

*DLCT* Dual-layer CT, *IC* Iodine concentration, *AP* Arterial phase, *VP* Venous phase, *NIC* Normalized iodine concentration, *LN* Lymph node, *λ*_*HU*_ Spectral curve slope.

Maximum standardized uptake values (SUV_max_) for all LNs that matched with sites on CT images were independently measured by two nuclear medicine physicians (abovementioned) by placing round 3D ROIs, which included the boundary of the entire lymph node (Fig. 2). The average values measured by two nuclear medicine physicians were considered the mean values (Fig. 3).

### Statistical analysis

SPSS 22.0 (IBM, Armonk, NY, USA), R software and MedCalc were used to perform statistical analysis. Continuous variables were presented as mean ± standard deviation; those with normal and skewed distributions were compared by the t-testand the Mann–Whitney U test between the LN-negative and LN-positive groups, respectively. The Chi-square test was used to compare categorical variables. *p* < 0.05 was considered statistically significant. Univariate and multivariate analyses were performed to determine independent risk factors for LN positivity. Odds ratios (ORs) with 95% confidence intervals (CIs) were used to quantitate the associations of morphologic and quantitative CT imaging parameters with LN positivity. The lowest value was 0, which indicated that the parameter was not predictive of LN positivity. A multivariate logistic regression model with tenfold cross-validation was used to build the best predictive model for differentiating LN positivity from LN negativity in the training cohort. Receiver operating characteristic (ROC) curves were plotted and areas under the curves (AUCs) were calculated to determine the diagnostic efficiencies of quantitative DLCT parameters and the predictive model for distinguishing negative and positive LNs, and differences in diagnostic performances were analyzed by the DeLong test^43^ in the training cohort. The AUCs of the best parameters or the predictive model were verified in the validation cohort. The inter-reader measurement repeatability of CT imaging parameters in LNs was evaluated in terms of intraclass correlation coefficient (ICC), with ICC > 0.75 considered to indicate reliable and stable repeatability (r = 1.0, perfect agreement; 0.81–0.99, almost perfect agreement; 0.61–0.80, substantial agreement; 0.41–0.60, moderate agreement; 0.21–0.40, fair agreement; and ≤ 0.20, slight agreement).

### Ethics approval and consent to participate

This retrospective study was approved by the Medical Ethics Committee of Tongji Medical College, Huazhong University of Science and Technology. Written informed consent was waived by the Institutional Review Board.

### Statement

All methods were carried out in accordance with relevant guidelines and regulations.

## Data Availability

The datasets used and/or analyzed during the current study are available from the corresponding author upon reasonable request.

## Abbreviations

AP: Arterial phase
CT: Computed tomography
DLCT: Dual-layer CT
18F-FDG PET/CT: 18F-fluorodeoxyglucosepositron emission tomography/computed tomography
IC: Iodine concentration
LD: Long-axis diameter
LN: Lymph node
NIC: Normalized iodine concentration
ROC: Receiver operating characteristic
SUV_max_: Maximum standardized uptake value
VP: Venous phase
λ_HU_: Spectral curve slope
